# Evaluation of the Immunomodulatory Properties of *Streptococcus suis* and Group B *Streptococcus* Capsular Polysaccharides on the Humoral Response

**DOI:** 10.3390/pathogens6020016

**Published:** 2017-04-20

**Authors:** Cynthia Calzas, Morgan Taillardet, Insaf Salem Fourati, David Roy, Marcelo Gottschalk, Hugo Soudeyns, Thierry Defrance, Mariela Segura

**Affiliations:** 1 Swine and Poultry Infectious Diseases Research Center (CRIPA), Department of Pathology and Microbiology, Faculty of Veterinary Medicine, University of Montreal, 3200 Sicotte St., Saint-Hyacinthe, QC J2S 2M2, Canada; cynthia.calzas@umontreal.ca (C.C.); dav.roy@gmail.com (D.R.); marcelo.gottschalk@umontreal.ca (M.G.); 2 CIRI, INSERM, U1111, CNRS UMR5308, University of Lyon 1, 21 Avenue Tony Garnier, 69007 Lyon, France; morgan.taillardet@inserm.fr (M.T.); thierry.defrance@inserm.fr (T.D.); 3 Department of Microbiology, Infectiology and Immunology, Faculty of Medicine, University of Montreal, C.P. 6128, Succ. Centre-ville, Montreal, QC H3C 3J7, Canada; insaf.salem@umontreal.ca (I.S.-F.); hugo.soudeyns@umontreal.ca (H.S.)

**Keywords:** *Streptococcus suis*, group B *Streptococcus*, capsular polysaccharide, sialic acid, humoral response, murine B cells

## Abstract

*Streptococcus suis* and group B *Streptococcus* (GBS) are encapsulated streptococci causing septicemia and meningitis. Antibodies (Abs) against capsular polysaccharides (CPSs) have a crucial protective role, but the structure/composition of the CPS, including the presence of sialic acid, may interfere with the generation of anti-CPS Ab responses. We investigated the features of the CPS-specific Ab response directed against *S. suis* serotypes 2 and 14 and GBS serotypes III and V after infection or immunization with purified native or desialylated CPSs in mice. Whereas *S. suis*-infected mice developed a very low/undetectable CPS-specific IgM response, significant anti-CPS IgM titers were measured in GBS-infected animals (especially for type III GBS). No isotype switching was detected in *S. suis*- or GBS-infected mice. While the expression of sialic acid was essential for the immunogenicity of purified GBS type III CPS, this sugar was not responsible for the inability of purified *S. suis* types 2, 14 and GBS type V CPSs to induce a specific Ab response. Thus, other biochemical criteria unrelated to the presence of sialic acid may be responsible for the inaptitude of the host immune system to mount an effective response against certain *S. suis* and GBS CPS types.

## 1. Introduction

*Streptococcus suis* and *Streptococcus agalactiae* (also known as group B *Streptococcus* (GBS)) are two encapsulated bacteria that induce similar pathologies, including septicemia and meningitis, in animals and/or humans. *S. suis* is a major pig pathogen, responsible for important economic losses in the swine industry, as well as an emerging zoonotic pathogen in humans, responsible for deadly outbreaks in Asian countries. GBS is a leading cause of life-threatening invasive bacterial infections in neonates and pregnant women, as well as in the elderly and immunocompromised individuals. Among the 35 *S. suis* and 10 GBS serotypes identified, *S. suis* types 2 and 14 and GBS types III and V are the most virulent and frequently isolated [1,2].

For both pathogens, the capsular polysaccharide (CPS), which defines the serotype, is considered as a major virulence factor that protects the bacteria against host immune responses [3,4,5]. However, the interplay of CPS with components of the innate immune system, including antigen-presenting cells (APCs), seems to differ radically between these two streptococci. Experiments using nonencapsulated mutants have shown that, in contrast to GBS CPS, *S. suis* CPS has a strong antiphagocytic effect and severely interferes with the activation and maturation of APCs [6,7,8,9,10,11]. Whereas the structures of GBS types III and V CPSs have been determined in the beginning of the 1990s [12,13], the structures of *S. suis* types 2 and 14 CPSs have only recently been elucidated [14,15]. The four CPSs are composed of glucose, galactose and *N*-acetylglucosamine and share structural features, being arranged into a repeating unit that contains a side chain terminated by sialic acid (*N*-acetylneuraminic acid). Remarkably, the presence of capsular sialic acid is a unique characteristic of *S. suis* and GBS among Gram-positive bacteria. Despite biochemical similarities, each CPS is made up of a unique arrangement of these sugars, conferring a distinct antigenicity. In addition, sialic acid forms an α-2,6 linkage with the adjacent galactose in *S. suis*, in contrast to the α-2,3 linkage found in GBS. Sialic acid of bacterial polysaccharides has been suggested to be involved in immune evasion mediated by molecular mimicry and by inhibition of complement activation, and this is possibly associated with the nature of sialic acid linkages [16,17,18,19]. Thus, the differential expression of sialic acid between *S. suis* and GBS might differentially modulate host immune responses.

The role of the humoral immunity and CPS-specific antibodies (Abs) in host defense against encapsulated bacteria is well established [20,21]. The efficacy of the protection of the different immunoglobulin (Ig) classes during bacterial infection is dependent on their affinity with cognate antigen and on their biological functions. IgG is particularly effective at mediating bacterial elimination by favoring bacterial opsonophagocytosis and/or by triggering the complement cascade directly at the surface of the pathogen [22,23]. In the case of *S. suis* and GBS, Abs directed against the CPS mediate protection in opsonophagocytosis assays and in vivo after passive transfer to animals before challenge [24,25,26]. Paradoxically, the cellular and molecular processes that lead to the development of CPS-specific Ab responses remain still elusive. By their inaptitude to recruit T cells during humoral responses, categorizing them as “thymo-independent” (TI) antigens, purified CPSs are usually less immunogenic than proteins [27,28]. Via their repeating epitopes, TI antigens are able to deliver strong and sustained intracellular signaling through multivalent membrane Ig cross-linking at the surface of specific B cells, resulting in an efficient and robust cell proliferation [27,28]. However, the engagement of the B cell receptor by TI antigens is not sufficient to induce B cell activation, and a second signal is required, which may be provided by APCs via the release of B cell-activating factor of the tumor necrosis factor family (BAFF). In the absence of T cells, this cytokine promotes Ig class switching in naive B cells and their terminal differentiation into plasma cells [29]. Mice deficient in BAFF or its receptors display an abrogated IgG response specific to the synthetic TI model antigen, nitrophenol (NP)-Ficoll [30].

Ligands of Toll-like receptors (TLRs) may also potentiate TI Ab responses. The engagement of TLR4 by LPS stimulates expression and production of BAFF by APCs, including dendritic cells (DCs) [29,31]. In addition, TLR9 agonists CpG oligodeoxynucleotides (ODNs) induce an increased expression of BAFF receptors by B cells [32]. However, the effectiveness of TLR ligands as adjuvants seems to depend on the nature of the TI antigen. For example, in mice, whereas CpG ODNs significantly increase the IgM and IgG responses to NP-Ficoll [32,33] and to purified *Streptococcus pneumoniae* type 3 CPS (PS3) [34], it does not heighten the immunogenicity of purified *S. pneumoniae* types 6B, 19F and 23F CPSs [33].

Some studies suggest that CPSs may also have intrinsic immunosuppressive properties. For example, *Neisseria meningitidis* group C CPS or certain types of pneumococcal CPSs induce hyporesponsiveness after immunization [35,36]. These bacterial CPSs were shown in vitro to inhibit the maturation and the pro-inflammatory activities of human macrophages and/or DCs and polarize immune responses toward a regulatory profile [37,38]. We have previously shown that purified *S. suis* and GBS CPSs partially inhibit BAFF expression in murine DCs [39]. However, it is unclear how these properties of CPSs influence the generation of the humoral immunity.

The goal of this study was to evaluate the influence of the CPS biochemistry on the development of the humoral response by comparing the features of CPS-specific Ab responses against *S. suis* types 2, 14 and GBS types III and V in mice. We first determined the characteristics of this response after a clinical infection with live virulent strains and after immunization with purified CPSs. We then investigated the in vitro interactions of each purified CPS with murine B cells and soluble factors involved in TI Ab responses, including CpG ODNs and BAFF. Finally, the influence of sialic acid was analyzed using chemically-desialylated CPSs.

## 2. Results

### 2.1. Distinct Features of the CPS-Specific Ab Response between Mice Infected with Live S. suis and GBS

In order to compare the features of the humoral immunity against *S. suis* and GBS CPSs, we first infected mice with live virulent strains of *S. suis* types 2 or 14 or GBS types III or V and evaluated the kinetics, magnitude and isotype profile of the induced anti-CPS Ab response. The protein-specific Ab response was also measured in parallel for each bacterial strain. Bacteremia was detected in all surviving mice at 12-h post-infection, ranging from ~10^4^ to 10^7^ CFU/mL for *S. suis* types 2 and 14, from 10^2^ to 10^6^ CFU/mL for GBS type III and from 10^2^ to 5 × 10^3^ CFU/mL for GBS type V, in accordance with previous studies [5,40,41] (Figure S1).

**Figure 1:**
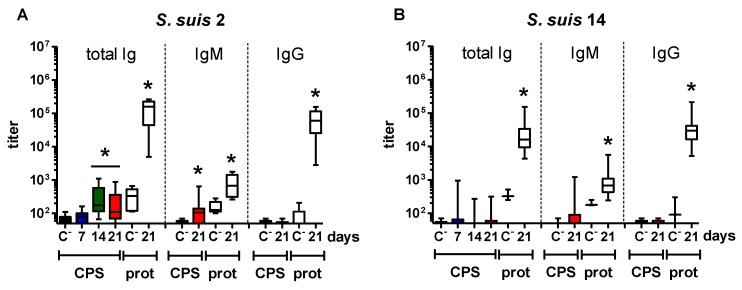

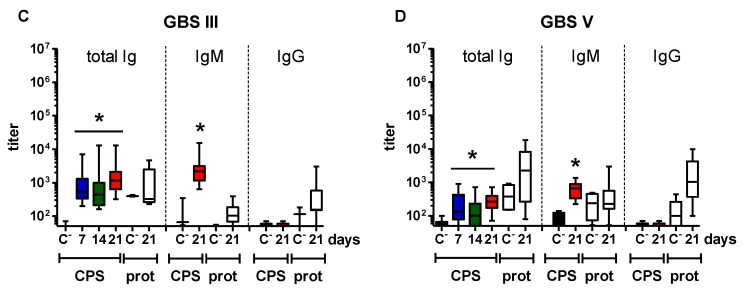
Titration of capsular polysaccharide (CPS)-specific antibodies in mice after infection with *S. suis* serotype 2, *S. suis* serotype 14, group B *Streptococcus* (GBS) serotype III or GBS serotype V. Mice were infected with 2 × 10^7^ CFU of live *S. suis* serotype 2 strain P1/7 (*n* = 60) (**A**); 5 × 10^6^ CFU of live *S. suis* serotype 14 strain DAN13730 (*n* = 40) (**B**); 2 × 10^6^ CFU of live GBS serotype III strain COH-1 (*n* = 40) (**C**); or 10^4^ CFU of live GBS serotype V strain CJB111 (*n* = 40) (**D**). Total Ig (IgG plus IgM) anti-CPS titers were determined by ELISA on Days 7 (in blue), 14 (in green) and 21 (in red) in surviving mice. IgM and IgG anti-CPS titers were determined on Day 21 (in red). For comparative purpose, total Ig (IgG plus IgM), IgM and IgG anti-protein (‘prot’) titers were also determined on Day 21. Data are presented in a box-and-whiskers diagram with the ends of whiskers representing the minimum and the maximum value. “C^−^” represents a pool of control mice (*n* = 3) injected with vehicle solution, whose titers were evaluated on Days 7, 14 and 21. * Statistically significant difference (*p* < 0.05) in comparison to the respective C^−^ group.

Mice infected with *S. suis* types 2 and 14 developed a significant anti-protein Ab response that was mainly composed of IgG, with an almost 300-fold increase in titers versus non-infected mice (Figure 1A,B). In contrast to the protein response, either a weak anti-CPS IgM response was observed from Day 14 to Day 21 post-infection in *S. suis* type 2-infected mice; or an almost complete absence of significant anti-CPS titers was noticed in *S. suis* type 14-infected animals (Figure 1A,B). There was a positive correlation between the anti-protein Ab titers and the bacteremia in mice infected with *S. suis* type 2 or 14, but not between the anti-CPS titers and the bacteremia in *S. suis* type 2-infected mice [41] (data not shown).

In opposition to *S. suis*-infected mice, GBS-infected animals presented a significant CPS-specific Ab response as soon as Day 7, which remained stable until Day 21 (Figure 1C,D). However, in GBS type V-infected mice, the magnitude was also relatively low, with an eight-fold increase in IgM titers compared to control mice. On the other hand, GBS type III-infected mice showed a 20-fold increase in anti-CPS IgM titers; though, no IgG anti-CPS titers were detected in GBS-infected mice (Figure 1C,D). Surprisingly, even if some GBS type III- or GBS type V-infected mice exhibited a significant protein-specific Ab response, this response was globally low. It should be noted that higher GBS bacterial doses were lethal early after infection and thus could not be evaluated. No correlation was found between the anti-CPS Ab titers and the bacteremia in GBS-infected mice. The low levels of Abs and/or blood bacteremia might preclude a positive correlation, which might vary upon the infection model.

### 2.2. Features of the CPS-Specific Ab Response in Mice Immunized with Purified S. suis and GBS CPSs

**Figure 2:**
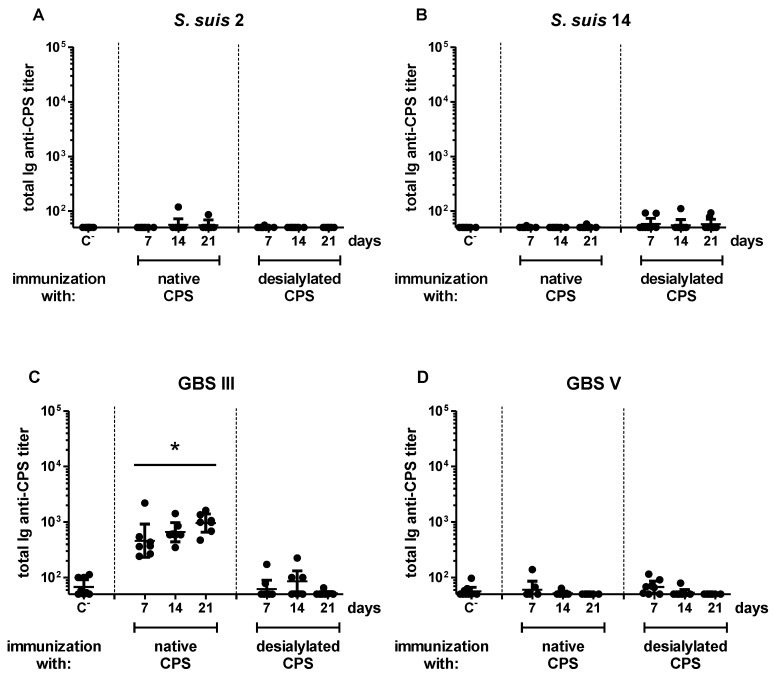
Titration of CPS-specific antibodies in mice after immunization with purified native or desialylated *S. suis* serotype 2, *S. suis* serotype 14, GBS serotype III or GBS serotype V CPS. Mice (*n* = 8) were immunized with 2 µg of purified native or desialylated *S. suis* serotype 2 (**A**); *S. suis* serotype 14 (**B**); GBS serotype III (**C**); or GBS serotype V (**D**) CPS emulsified with STIMUNE^®^. Total Ig (IgG plus IgM) anti-native CPS titers were determined by ELISA on Days 7, 14 and 21. “C^−^” represents a pool of control mice (*n* = 3) injected with STIMUNE^®^ only, whose titers were evaluated on Days 7, 14 and 21. Data from individual mice are presented, including the geometric mean with 95% confidence interval. * Statistically significant difference (*p* < 0.05) in comparison to the C^−^ group.

A live bacterium contains numerous protein and polysaccharide antigens that are secreted and/or intimately co-expressed within an organized particulate structure owning multiple adjuvanting moieties. This may confer unique immunogenic properties to the CPS expressed in the context of an intact bacterium [21]. In order to directly evaluate the influence of the CPS biochemistry on the development of the humoral immunity against *S. suis* and GBS CPSs, we then immunized mice with purified *S. suis* type 2 or 14 or GBS type III or V CPS and compared the features of the induced primary anti-CPS Ab response (Figure 2).

Like with live bacteria, the specific Ab response in mice immunized with *S. suis* type 14 CPS remained similar to that of the negative control group throughout the experiment (Figure 2B). Unlike mice infected with live *S. suis* type 2 or GBS type V, anti-CPS Ab titers were no longer measured in mice immunized with the CPS purified from these two pathogens (Figure 2A,D). In contrast, we observed a significant response in mice immunized with purified CPS from GBS type III, with similar amplitude and kinetics to what was monitored after infection with the bacteria (Figure 2C). This response was composed of IgM, and no IgG was detected (Figure S2). In accordance with the inability of purified TI antigens to generate an amplified reaction subsequent to a second administration [42], no significant difference in the kinetics, magnitude or isotype profile was observed between primary and secondary specific Ab responses against each of the four CPSs (Figure S3). Therefore, whereas the presence of subcapsular and/or secreted bacterial components may be required for the generation of *S. suis* type 2 and GBS type V CPS-specific Abs in mice, GBS type III CPS possesses intrinsic immunogenic properties. *S. suis* type 14 CPS has no immunogenic potential, even when associated with the bacterial surface.

### 2.3. Influence of Sialic Acid on the Features of the CPS-Specific Ab Response in Mice Immunized with Purified S. suis and GBS CPSs

**Figure A1:**
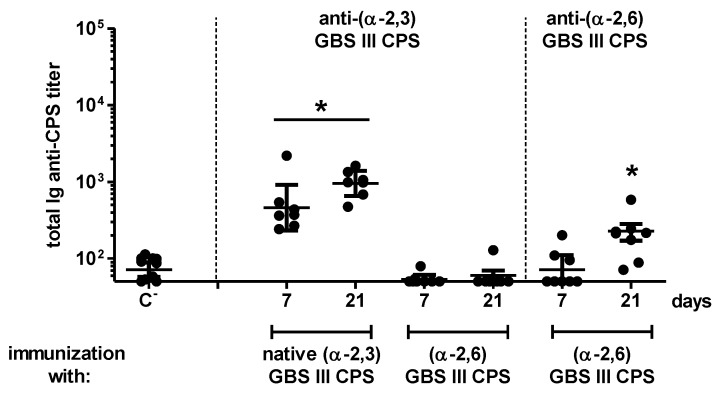
Titration of CPS-specific antibodies in mice after primary immunization with (α-2,3) or (α-2,6) GBS serotype III CPS. Mice (*n* = 8) were immunized with 2 µg of (α-2,3) or (α-2,6) GBS serotype III CPS emulsified with STIMUNE^®^ on Day 0. Total Ig (IgG plus IgM) anti-(α-2,3) (left) or anti-(α-2,6) (right) GBS serotype III CPS titers were determined by ELISA on Days 7 and 21. “C^−^” represents a pool of control mice (*n* = 3) injected with STIMUNE^®^ only, whose anti-(α-2,3) or anti-(α-2,6) GBS serotype III CPS titers were evaluated on Days 7 and 21. Data from individual mice are presented, including the geometric mean with 95% confidence interval. * Statistically significant difference (*p* < 0.05) in comparison to the C^−^ group.

The expression of sialic acid by pathogens interferes with the host immunity by preventing complement activation on microbial surfaces [16,17], as well as by binding to inhibitory receptors expressed by immune cells [18,19]. However, the consequences of sialic acid expression on the development of humoral responses remain poorly explored. As the deletion of genes involved in sialic acid synthesis results in considerable or complete loss of CPS expression by *S. suis* and GBS [5,43,44], the influence of this sugar has been evaluated by using purified, chemically-desialylated CPSs. As shown in Figure 2 and Figure S3, no significant difference in the CPS-specific Ab response was observed between mice immunized with native and desialylated *S. suis* type 2, *S. suis* type 14 or GBS type V CPS. In contrast, a significant decrease in GBS type III CPS-specific Ab response was detected in mice immunized with desialylated CPS, with Ab titers returning to basal levels of the placebo group (Figure 2C and Figure S3C). This is in accordance with previous studies suggesting that sialic acid exerts a conformational control of the helical structure of the immunodominant epitope of this CPS [45]. To test this hypothesis and further evaluate the impact of the sialic acid α-2,3 linkage vs. α-2,6 linkage, we generated α-2,6 sialylated GBS type III CPS (see Appendix A). Compared to native (α-2,3) CPS, the (α-2,6) CPS completely lost its immunogenic capacity as a non-significant Ab response against the native CPS could be detected in mice immunized with this modified polysaccharide (Figure A1, left panel). Nevertheless, a low albeit significant Ab response against the α-2,6 modified form itself was observed, suggesting the generation of a new epitope with no cross-reaction with the native form (Figure A1, right panel). Therefore, whereas the presence of sialic acid may not exert a major modulatory effect on the poor ability of purified *S. suis* types 2, 14 and GBS type V CPSs to induce a specific Ab response in mice, the expression of this sugar (in its α-2,3 linkage) is essential for the immunogenicity of GBS type III CPS.

### 2.4. In Vivo Effect of Exogenous TLR Agonists on S. suis Type 2 CPS-Specific Ab Response

**Figure 3:**
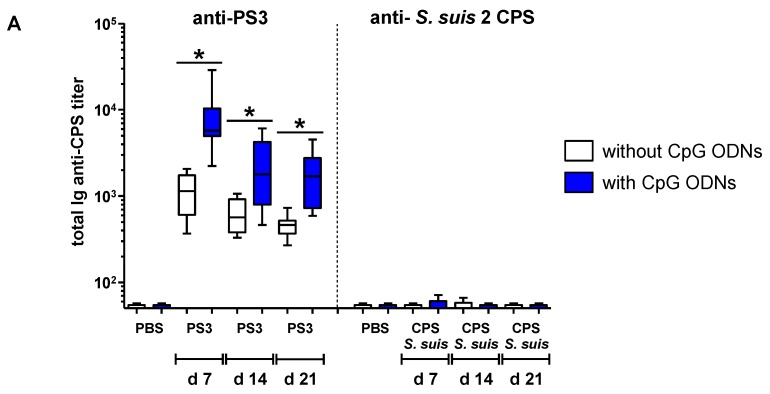

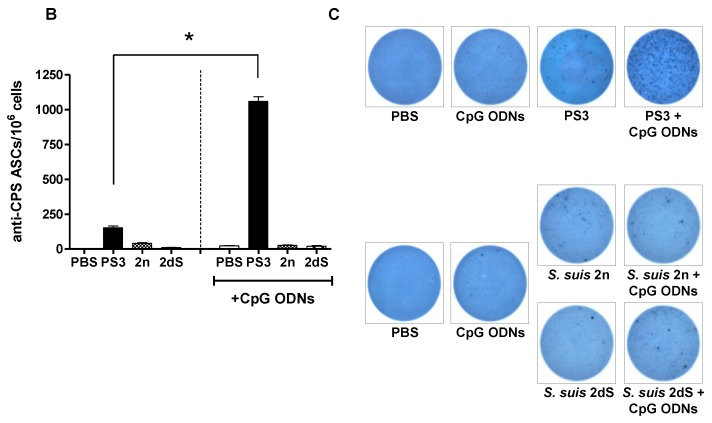
Adjuvant effect of CpG oligodeoxynucleotides (ODNs) on the CPS-specific humoral response in mice immunized with purified native or desialylated *S. suis* serotype 2 CPS or purified *S. pneumoniae* serotype 3 CPS (PS3). (**A**) In a first set of experiments, mice (*n* = 8) were immunized with 2 µg of purified native (n) or desialylated (dS) *S. suis* serotype 2 CPS (CPS *S. suis*) or PS3 in PBS on Day 0 and 80 µg of CpG ODNs two days after. The control group (*n* = 8) received CPS or PS3 on Day 0 and PBS two days later. The placebo group (*n* = 3) received PBS or CpG ODNs only. Total Ig (IgG plus IgM) anti-native *S. suis* type 2 CPS or anti-PS3 titers were determined by ELISA on Days 7, 14 and 21. Data are presented in a box-and-whiskers diagram with the ends of whiskers representing the minimum and the maximum value. (**B**) In a second set of experiments, mice (*n* = 5) were immunized as described in (A), but splenocytes were collected on Day 5 after immunization, and anti-CPS antibody-secreting cells (ASCs) were enumerated by ELISpot as described in the Materials and Methods section. Data are expressed as arithmetic means with SEM. (**C**) Visualization of PS3 (top) or native *S. suis* type 2 CPS (bottom) specific ASCs in ELISpot wells from splenocytes of mice obtained in (B). * *p* < 0.05 between “PS3” and “PS3 + CpG ODNs” groups.

In comparison with *S. suis* type 2 CPS expressed in the context of an intact bacteria, the complete loss in the immunogenicity of the same antigen administrated under a purified form suggests that bacterial non-capsular factors could potentiate the development of CPS-specific humoral response. We thus evaluated if an exogenous TLR agonist, expected to mimic some of the co-signals provided by whole bacteria, could modulate the magnitude of the Ab response directed to purified *S. suis* type 2 CPS (selected as a model CPS for the subsequent study). We focused our work on CpG ODNs because previous studies have demonstrated that these TLR9 agonists amplified the humoral reaction against purified bacterial CPS, provided that they were administrated a few days after immunization with CPS [34,46]. Nevertheless, following the same experimental protocol, no improvement of the *S. suis* type 2 CPS-specific serum Ab response was obtained when CpG ODNs were injected into mice, and Ab titers remained undetectable from Day 7 to Day 21 (Figure 3A).

This was in sharp opposition with the significant adjuvant effect observed on total Ig (IgG plus IgM) PS3-specific serum Ab response during the same period (Figure 3A). Like in previous reports [34,46], CpG ODNs promoted a rise of both IgM and IgG anti-PS3 titers (Figure S4). The inability of CpG ODNs to potentiate *S. suis* type 2 CPS-specific Ab response may not be related to the expression of sialic acid, as a similar result was obtained in mice immunized with desialylated CPS plus CpG ODNs (Figure 3A). In accordance with the results obtained with serum Ab titers, the injection of CpG ODNs significantly increased the number of splenic anti-PS3 Ab-secreting cells (ASCs), whereas specific ASCs were not significantly detected in the spleen of mice immunized with native or desialylated *S. suis* type 2 CPS, whether mice received CpG ODNs or not (Figure 3B,C).

### 2.5. In Vitro Study of the Immunomodulatory Effect of Purified S. suis and GBS CPSs

**Figure 4:**
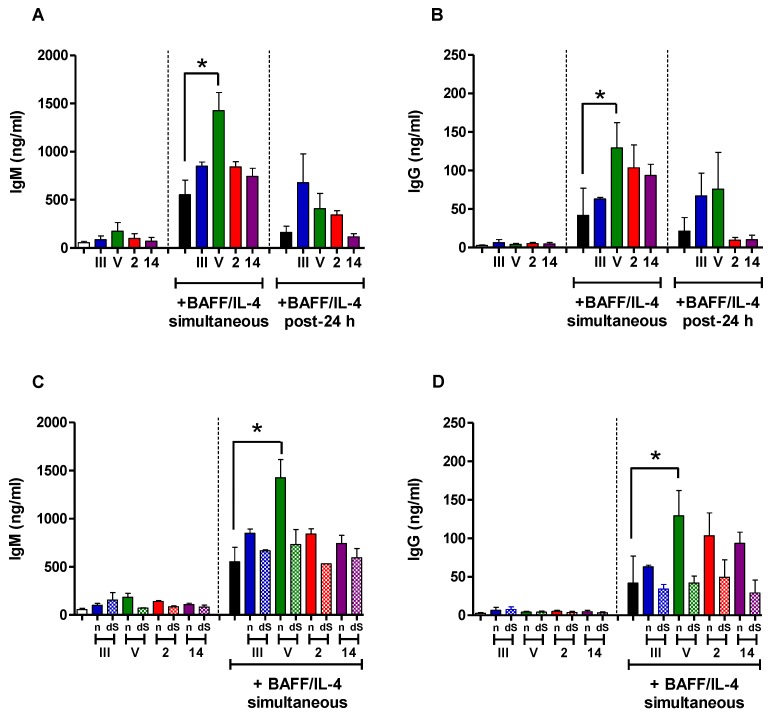
In vitro immunomodulatory effect of purified native or desialylated *S. suis* serotype 2, *S. suis* serotype 14, GBS serotype III or GBS serotype V CPS on BAFF/IL-4-induced Ig secretion by naive B cells. (**A**,**B**) Mouse splenic B cells (10^6^ cells/mL) were incubated with purified native *S. suis* serotype 2, *S. suis* serotype 14, GBS serotype III or GBS serotype V CPS (each at 20 µg/mL) simultaneously with (central panel) or 24 h before the addition of (right panel) BAFF (1 µg/mL) along with IL-4 (50 ng/mL); (**C**,**D**) in order to evaluate the influence of sialic acid, cells were also incubated with desialylated (dS) *S. suis* serotype 2, *S. suis* serotype 14, GBS serotype III or GBS serotype V CPS in parallel to cells incubated with the respective native CPS (n) as described in (**A**,**B**). After seven days of incubation, supernatants were collected, and total IgM (**A**,**C**) and IgG (**B**,**D**) was quantified by ELISA. Cells stimulated with BAFF/IL-4 alone (black bars) served as a positive control. In the left panel, cells stimulated with medium (white bars) or each purified CPS alone are represented as an indication of the basal Ig secretion level of cells in absence of BAFF/IL-4 stimulation. Data are expressed as arithmetic means with the SEM of three (central and right panels) or four (left panels) experiments. * *p* < 0.05.

In order to get a better understanding of the cellular and molecular mechanisms responsible for the poor immunogenicity of *S. suis* types 2 and 14 and GBS type V CPSs, as well as the relatively higher ability of GBS type III CPS to induce a specific Ab response, we compared the in vitro interactions of each purified CPS with naive B cells isolated from mouse spleen, a central organ in the implementation of Ab reaction against TI antigens [47,48]. In particular, we evaluated the immunomodulatory effect of CPSs on the capacity of B cells to secrete Ig in response to soluble factors critically involved in the generation of TI humoral immunity. We first assessed the influence of CPS on the ability of BAFF to promote Ig secretion by B cells. Stimulation with BAFF along with IL-4 was required for Ig production, and cells incubated with media or each CPS alone secreted very low levels of IgM or IgG (Figure 4, left panels).

**Figure 5:**
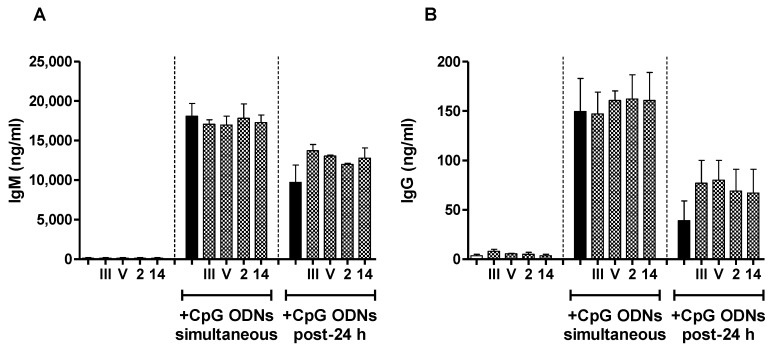
In vitro immunomodulatory effect of purified native *S. suis* serotype 2, *S. suis* serotype 14, GBS serotype III or GBS serotype V CPS on the Ig secretion by naive B cells induced by CpG ODNs. Mouse splenic B cells (10^6^ cells/mL) were incubated with purified native *S. suis* serotype 2, *S. suis* serotype 14, GBS serotype III or GBS serotype V CPS (each at 20 µg/mL) simultaneously with (central panel) or 24 h before the addition of (right panel) CpG ODNs (1 µg/mL). After seven days of incubation, supernatants were collected and total IgM (**A**) and IgG (**B**) were quantified by ELISA. Cells stimulated with CpG ODNs alone (black bars) served as the positive control. In the left panel, cells stimulated with medium (white bars) or each purified CPS alone are represented as an indication of the basal Ig secretion level of cells in absence of stimulation by CpG ODNs. Data are expressed as arithmetic means with the SEM of three (central and right panels) or four (left panel) experiments.

There was an overall tendency to an increase of the Ig production by B cells incubated with the CPS plus BAFF/IL-4 in comparison with cells incubated with BAFF/IL-4 only, whether CPS was added 24 h before or simultaneously to BAFF/IL-4, and this seemed to be related to the presence of sialic acid. We then evaluated the effect of *S. suis* and GBS CPSs on the Ig secretion by B cells induced by CpG ODNs. The addition of the different CPSs (either native or desialylated) did not influence the ability of CpG ODNs to bring about IgM or IgG production by B cells, and a similar conclusion was obtained when CPS was added 24 h before or simultaneously to CpG ODNs (Figure 5 and Figure S5).

Therefore, in our hands, the poor immunogenicity of *S. suis* CPSs and GBS type V CPS could not be explained by the induction of an immunosuppressive state of B cells, making them unreactive to the stimulation by BAFF/IL-4 or CpG ODNs.

### 2.6. Study of the Immunomodulatory Effect of Purified S. suis and GBS CPSs on a T Cell-Dependent Response

**Figure 6:**
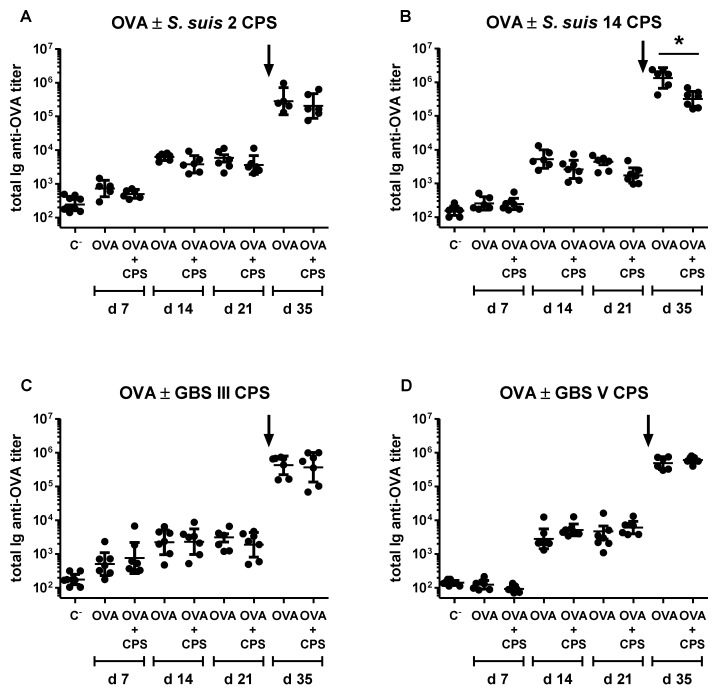
In vivo immunomodulatory effect of purified native *S. suis* serotype 2, *S. suis* serotype 14, GBS serotype III or GBS serotype V CPS on ovalbumin (OVA)-specific antibody response. Mice (*n* = 8) were co-immunized intraperitoneally with 10 µg of OVA and 2 µg of purified native *S. suis* serotype 2 (**A**), *S. suis* serotype 14 (**B**), GBS serotype III (**C**) or GBS serotype V (**D**) CPS in PBS on Days 0 and 21. Control group (*n* = 8) received OVA only on Days 0 and 21. Total Ig (IgG plus IgM) anti-OVA titers were determined by ELISA on Days 7, 14, 21 and 35. “C^−^” represents a pool of placebo mice (*n* = 3) injected with PBS, whose titers were evaluated on Days 7, 14, 21 and 35. Data from individual mice are presented, including the geometric mean with 95% confidence interval. An arrow indicates secondary immunization. * *p* < 0.05.

In the light of the reported immunosuppressive effect of encapsulated bacteria on the generation of humoral immunity specific to heterologous proteins [49], as well as the ability of purified microbial polysaccharides to interfere with the presentation of T cell-dependent (TD) antigens by APCs [50], we assessed if purified *S. suis* and GBS CPSs could prevent the development of ovalbumin (OVA)-specific Ab response in mice. We chose this TD antigen model because bacterial CPSs have been previously described to impede the activation of OVA-specific T cells [51]. As shown in Figure 6, the co-injection of OVA with *S. suis* type 2 or 14 or GBS type III or V CPS did not hamper the generation of primary OVA-specific Ab response.

Except for a slight inhibitory effect of *S. suis* type 14 CPS, no major influence of *S. suis* or GBS CPS was noticed on the generation of the memory OVA-specific Ab response. Similar results were obtained regardless of the concentration of CPS (2 or 20 µg) and the route of immunization (s.c. or i.p.) (data not shown).

## 3. Discussion

The humoral response is the main mechanism of host adaptive immunity in the fight against infections with extracellular pathogens. Via their biological functions of opsonophagocytosis, CPS-specific Abs allow clearance of bacteria by the immune system. This is the rationale for the development of vaccines against *S. pneumoniae*, *N. meningitidis* and *Haemophilus influenzae* infections in humans [20,21]. Despite being an important pig pathogen and an emerging threat to human health, no efficient vaccine against *S. suis* is currently available [52]. Whole-cell killed bacterins used in the field provide limited protection, and experimental live-attenuated vaccines have been tried with contradictory results and the inherent risk of zoonosis. Moreover, the high genotypic and phenotypic variations among *S. suis* strains of different geographical origins may preclude the use of protein subunit vaccines [52]. Concerning GBS, despite the fact that protein-based vaccines confer potent defense in clinical trials, they generally do not provide broad-coverage protection [53]. For both *S. suis* and GBS, the CPS represents a target of choice, not only because CPS-specific Abs display a good protective potential in experimental studies [24,25,26], but also because a vaccine composed of CPSs purified from several serotypes would be the key to a universal vaccine. However, the design of CPS-based vaccines is an area of research only recently explored for *S. suis* [54], and the efficacy of in-trial GBS vaccines depends on the serotype of the CPS included in the preparations [26,55]. Indeed, CPSs of types Ia, Ib, II and III induce strong protective IgG responses, whereas GBS type V CPS promotes higher concentrations of specific IgM than IgG. Yet, the mechanisms for the generation of *S. suis* and GBS CPS-specific Ab responses, as well as the influence of intrinsic immunomodulatory properties of CPS on its immunogenicity have been poorly explored. Our work is the first to compare, within the same study, the impact of the composition and/or structure of *S. suis* types 2, 14 and GBS types III and V CPSs on the generation of humoral responses, including the influence of the presence of sialic acid.

Bacterial CPSs are classically encountered by the host immune system covalently attached to the underlying subcapsular domain. We thus initially compared the anti-CPS Ab response subsequent to a clinical infection with live virulent *S. suis* type 2 or 14 or GBS type III or V in mice. Our results showed that CPS-specific Ab responses to whole GBS or *S. suis* exhibit typical features of a TI reaction in spite of CPS association with the bacterial surface. It is interesting to note that similar findings, in terms of poor anti-CPS antibody response, were observed with other *S. suis* serotype 2 strains [41,56], suggesting that the characteristics of the anti-CPS antibody response do not seem to be influenced by the composition and/or architecture of the bacterial subcapsular domain, at least in the case of *S. suis*. Regardless of this common TI nature, infection with GBS type III resulted in a significant production of anti-CPS Abs, whereas a weak or negative humoral anti-CPS response was observed after infection with other bacterial types, especially for *S. suis*. The dissimilar CPS-specific Ab responses subsequent to infection with *S. suis* and GBS could be attributed to differences in the kinetics of bacterial multiplication, dissemination and survival in the host. In this regard, the total anti-protein response was also dissimilar between these two bacterial species. Previous studies demonstrated that the expression of CPS differently modulated the interactions of whole *S. suis* and GBS with the host innate immunity. Experiments using nonencapsulated mutant strains showed that *S. suis* types 2 and 14 CPSs crucially protected bacteria from phagocytosis by APCs, including DCs and macrophages [5,6,7,8,11]. Remarkably, *S. suis* type 2 CPS did not act simply as an inert physical barrier against phagocytes, but actively down-modulated signaling pathways involved in phagocytosis [8,57]. In contrast to *S. suis*, GBS types III and V were efficiently internalized by APCs [6,7,8,9,10,11]. Therefore, the different interplay of *S. suis* and GBS with innate immune cells during the course of infection may promote distinct adaptive immune reactions and may explain the disparate CPS- and protein-specific humoral response that were observed between the two pathogens. The different and globally-weak CPS-specific Ab responses subsequent to infection with *S. suis* and GBS could also be attributed to the immunomodulatory influence of secreted factors and/or non-capsular components expressed within the bacteria. In contrast to *S. suis* and GBS, it was reported that immunization with intact heat-killed *S. pneumoniae* capsular type 14 [58] or *N. meningitidis* serogroup C [59] induced significant IgM and IgG anti-CPS responses. The authors suggested that the nature of the in vivo anti-CPS response was markedly influenced by the composition and/or architecture of the bacterial subcapsular domain [60]. Using inactivated GBS type III immunization, not only a relatively high anti-CPS IgM response, but also modest IgG titers were observed [60]. This observation adds another level of complexity to the analysis of the features of the anti-CPS responses against whole bacteria, as live organisms might behave differently from inactivated ones. Therefore, the distinct CPS-specific Ab responses that we have observed in our experimental infections with live bacteria, such as the IgM-restricted isotype profile, might also be related to the influence of antigens that are selectively produced in vivo.

Several studies have suggested that the anti-CPS responses to intact bacteria versus isolated CPS are distinct [21]. For example, whereas purified *S. pneumoniae* type 14 CPS behaved as a TI antigen, its expression at the surface of intact bacteria markedly increased its immunogenicity in mice [58,61]. This included a newly-acquired capacity to recruit T cell help in the development of humoral response, displayed by high titers of CPS-specific IgG. In the present study, the anti-CPS response against *S. suis* type 2 and GBS type V (but not that of GBS type III) was absolutely dependent on CPS expression on the bacterial surface. In the case of purified *S. suis* types 2 and 14 CPSs, the complete lack of immunogenicity as a soluble antigen was observed at different doses (ranging from 1 to 25 µg) and despite the addition of different adjuvants [54] (unpublished observations). Of note, the TLR4 agonist monophosphoryl lipid A did not exert any adjuvant effect on *S. suis* type 2 CPS-specific Ab response in mice (Figure S6), despite the fact that this ligand has the capacity to restore the immunogenicity of a synthetic sialylated TI antigen [62]. Moreover, and in contrast to PS3 [34,46], the TLR agonists CpG ODNs were also unable to rescue soluble *S. suis* type 2 CPS immunogenicity. Co-immunization with purified PS3 and CpG ODNs enhanced the CPS-specific Ab response in mice, with similar efficiency and longevity as a PS3-tetanus toxoid conjugate vaccine [34,46]. It was demonstrated that CpG ODNs exerted their adjuvant effect directly on TI-specific B cell clones by promoting survival, proliferation and plasma cell differentiation [46]. Similar to our results, co-injection of mice with CpG ODNs and purified *H. influenzae* type b or *N. meningitidis* group C CPS did not improve the specific humoral response [63,64]. Thus, the Ab response to bacterial CPSs cannot be generalized as important inter-species differences exist. Furthermore, the immunogenicity of the CPS is affected by its presentation form (associated with the bacterial wall or soluble); though this effect also depends on the CPS type.

Besides secreted and/or closely co-expressed non-capsular antigens by the bacteria, intrinsic properties of the CPS itself may direct the development of CPS-specific Ab responses. One element that may play a crucial role is sialic acid. This sugar is widely expressed at terminal positions of glycoconjugates exposed at the surface of most mammalian leucocytes, and by interacting with sialic-acid binding lectins (Siglecs) expressed on the same cell (*cis* interaction), it maintains a constitutive inhibitory tone of immune cells [18,65,66,67,68]. Remarkably, sialylated bacteria can exploit these receptors to dampen immune responses [18,19]. However, our study suggests that sialic acid does not significantly modulate the generation of Ab response specific to *S. suis* and GBS CPSs. Furthermore, the presence of sialic acid in its native α-2,3 linkage is absolutely required for GBS type III CPS immunogenicity. In contrast to our results, chemical alteration of sialic acid of purified *N. meningitidis* group B CPS greatly improved immunogenicity of the CPS, including IgG isotype switching in mice [69]. A CPS-specific IgM-to-IgG isotype switching was also observed in macaques immunized with purified desialylated GBS type V CPS in comparison with animals receiving the native CPS [55]. However, a TD form of CPS (CPS conjugated to a carrier protein) was used in both studies, and thus, the participation of T cells in the development of the humoral response may explain the divergent conclusions with the present work. In this regard, modifications of sialic acid of unconjugated *N. meningitidis* group B CPS had no influence on its immunogenicity [69]. Variations in the composition of the repeating units unconnected to the presence of sialic acid, in the molecular weight of the CPS, as well as in the spacing, the rigidity and the multivalence of the epitopes of the CPS may then account for the different immunogenic properties of purified GBS type III versus *S. suis* types 2, 14 and GBS type V CPSs. It is interesting to note that the chemical modification of purified *S. suis* type 2 [54] and GBS type V CPSs [55] by a covalent conjugation to a carrier protein conferred to these molecules the ability to trigger a CPS-specific Ab response, indicating that the non-immunogenicity of these purified CPSs is not due to the absence of CPS-specific B cells in the repertoire of the host.

Purified B cell subsets preferentially involved in TI responses are particularly reactive to in vitro stimulation by TLR ligands [70]. In addition, these molecules induced BAFF production by innate immune cells [29,31], promoted the expression of BAFF receptors by B cells [32] and the co-engagement of TLRs and BAFF receptors synergized to improve B cell activation in the absence of T cell help [71]. As such, some evidence suggests that the poor immunogenicity of bacterial CPSs is related to their suppressive action on BAFF and its receptors [64]. Whereas we have previously demonstrated that purified *S. suis* types 2, 14 and GBS types III and V CPSs partially impaired in vitro BAFF expression by murine DCs [39], in the present study, we were unable to see a down-modulatory effect of these CPSs on the ability of exogenous BAFF to induce in vitro Ig secretion by murine B cells. Furthermore, we did not observe either an immunosuppressive or immunomodulatory effect of purified (either native or desialylated) *S. suis* CPS or GBS CPS on the Ig secretion by B cells mediated by CpG ODNs in our in vitro culture system. If these CPSs exhibit immunosuppressive effects, they may thus exert their action preferentially towards APCs and not B cells. Contrary to our results, in addition to blunting the production of BAFF by DCs, purified *N. meningitidis* group C CPS partially inhibited the in vitro response of murine B cells to BAFF stimulation [64]. The strong immunosuppressive effect of the *N. meningitidis* group C CPS can be explained by the fact that it is an α-2,9 linked homopolymer of sialic acid. The highest degree of presentation of sialic acid molecules by *N. meningitidis* group C CPS might result in an interaction of higher affinity with host Siglecs than *S. suis* and GBS CPSs. Variations in experimental conditions could also account for the observed differences, including the mouse strain origin and the subsets of B cells used. Indeed, our in vitro B cell culture model did not include all of the B cell subsets thought to be involved in TI Ab responses. In particular, B1 B cells, an important cellular population in polysaccharide-specific Ab responses [42,48], were counter-selected during the protocol of purification (these cells express the surface marker CD43), and we have focused our work on the interactions of *S. suis* and GBS CPSs with B2 B subsets (including both marginal zone and follicular B cells). However, both B1 and B2 subsets were affected by the immunosuppressive effect of *N. meningitidis* group C CPS [64], and in vivo tolerance induction by a sialylated polymer targeted, at least in part, follicular B cells [62]. The dose of the CPS might also influence its immunosuppressive effect, with higher doses being more suppressive, as previously reported [64]. Yet, in our hands, an increase in the CPS dose failed to reveal a modulatory effect in vitro (unpublished observations). Finally, we have no explanation for the apparent tendency of our CPS preparations to increase in vitro B cell responsiveness to BAFF, an effect that seems to be favored by the presence of sialic acid. This question warrants further investigations.

Overall, we have found that sialylated CPSs expressed from two distinct serotypes of two different Gram-positive streptococci, *S. suis* and GBS, are poorly-immunogenic antigens. The inability of host to mount an effective Ab response specifically directed against purified CPSs of these two pathogens do not seem to be explained by the capacity of sialic acid on its own to have instructed immune cells to recognize CPS as a “self-antigen” or to actively dampen B cell functions. This is further evidence by the lack of suppressive effect on a TD response in vivo (for instance, the co-injection of purified *S. suis* or GBS CPSs with OVA did not significantly dampen the generation of OVA-specific Abs in mice). Our results also suggest that terminal sialic acid of Gram-positive bacterial CPSs may not exert a marked inhibitory effect on the anti-CPS humoral response contrary to sialylated Gram-negative bacterial CPSs. Indeed, in the case of GBS type III, this sugar is part of the immunodominant epitope. The failure of intact bacteria, where CPS is associated with a particulate structure with numerous adjuvanting moieties, to generate an optimal anti-CPS response, including specific IgG production, might be due to intrinsic biochemical properties of these CPSs that could be related or not to the presentation of CPS on the bacterial surface. Their identification will be a valuable tool for a better understanding of the immunopathogenesis of *S. suis* and GBS infections, as well as for the development of efficient strategies to fight against these bacteria.

## 4. Materials and Methods

### 4.1. Mice

Female 5- to 8-week-old C57BL/6 mice (Charles River Laboratories) were acclimatized to standard laboratory conditions with free access to water and rodent chow. All experiments were conducted in accordance with the guidelines and policies of the Canadian Council on Animals Care and the principles set forth in the *Guide for the Care and Use of Laboratory Animals* by the Animal Welfare Committee of the University of Montreal (Protocol # 2016-Rech-1399 and 1523) [72].

### 4.2. Bacterial Strains and Growth Conditions

Encapsulated virulent *S. suis* serotype 2 strain P1/7 isolated from a pig with meningitis [73], *S. suis* serotype 14 strain DAN13730 isolated from a human with meningitis [74], GBS serotype III strain COH-1 isolated from an infant with sepsis and meningitis [75] and GBS serotype V strain CJB111 (ATCC BAA-23) isolated from a neonate with septicemia were used for experimental infections. These strains were already used in earlier studies and were cultured as previously described [5,10,40]. Briefly, bacteria were grown overnight onto sheep blood agar plates at 37 °C and isolated colonies were cultured in 5 mL of Todd–Hewitt broth (THB; Becton Dickinson, Mississauga, ON, Canada) for 8 h at 37 °C. Then, 10 µL of a 10^−3^ dilution of 8 h-cultures were transferred into 30 mL of THB and incubated for 16 h (*S. suis*) or 12 h (GBS) at 37 °C. Stationary phase bacteria were washed in phosphate-buffered saline (PBS, pH 7.3). The bacterial pellet was then resuspended in THB and adjusted to the desired concentrations. Non-encapsulated mutant strains Δ*cpsF* derived from strain P1/7, Δ*cps14b* derived from strain DAN13730 [5], Δ*cpsE* derived from strain COH-1 [9] and Δ*cpsE* derived from strain CJB111 [10] were used as coating for enzyme-linked immunosorbent assay (ELISA) of non-capsular, mainly protein-specific Ab response as described previously [41]. For both encapsulated and non-encapsulated strains, aliquots of final bacterial suspensions were plated using an Autoplate 4000 automated spiral plater (Spiral Biotech, Norwood, MA, USA) onto sheep blood agar plates (Oxoid, Nepean, ON, Canada), and colonies were accurately counted after overnight incubation at 37 °C.

### 4.3. CPS Purification and Desialylation

The native CPSs of *S. suis* type 2, *S. suis* type 14, GBS type III and GBS type V were purified as previously described [39]. Desialylated CPSs were obtained by mild acid hydrolysis [39]. Each purified CPS was subjected to rigorous physicochemical and immunologic quality control tests to ensure the identity and the purity of the CPS, the preservation of epitope recognition and the absence of sialic acid in the desialylated preparations [39]. Briefly, the absence of nucleic acid and protein contamination was confirmed by spectrophotometry; each CPS was analyzed by nuclear magnetic resonance (NMR), and the monosaccharide composition was confirmed by methanolysis followed by acetylation and analysis by gas chromatography, either with flame ionization detection or coupled to mass spectrometry. The presence or absence of sialic acid was verified by NMR and by an enzyme-linked lectin assay. Finally, the preservation of epitope recognition was confirmed by Dot-ELISA using CPS-specific sera, as previously described [39]. Similar yields of CPS production were obtained with the four strains per liter of culture under normalized O.D. _600nm_ values of 0.8. The average CPS yield was of 6.8 ± 2.6 mg/L for *S. suis* type 2, 6.4 ± 1.9 mg/L for *S. suis* type 14, 6.3 ± 4.2 mg/L for GBS type III, and 6.4 ± 1.5 mg/L for GBS type V.

### 4.4. Bacterial Infections

A live suspension of 2 × 10^7^ CFU of strain P1/7, 5 × 10^6^ CFU of strain DAN13730, 2 × 10^6^ CFU of strain COH-1 or 10^4^ CFU of strain CJB111 was administrated intraperitoneally (i.p.) to mice on Day 0. Optimal bacterial doses were determined in standardization pre-trials and vary depending on the pathogenicity of each strain (data not shown). Negative control mice were injected with vehicle solution (sterile THB). Blood bacterial loads were assessed by collecting a 5-µL blood sample from the tail of each mouse at 12 h post-infection. Proper dilutions were plated and bacterial numbers counted as described above. Samples were taken on Days 7, 14 and 21 post-infection. To reduce the number of mice, on Days 7 and 14, blood samples were collected from the tail vein of each mouse for anti-CPS Ig titration. On Day 21, mice were euthanized and bled for anti-CPS and anti-protein Ig titration. The numbers of animals included in all experiments are detailed in the figure legends.

### 4.5. Immunizations

In a first set of experiments aimed to compare the immunogenicity of purified *S. suis* types 2, 14 and GBS types III and V CPSs, as well as to evaluate the influence of the presence of sialic acid, mice were immunized subcutaneously (s.c.) twice at 3-week intervals with 2 µg of each native or desialylated CPS emulsified with STIMUNE^®^ (Prionics, La Vista, NE, USA) following the manufacturer’s recommendations, in a final volume of 100 µL. STIMUNE^®^ is a water-in-oil adjuvant composed of purified and defined mineral oil (Markol 52) with Span 85 and Tween 85 as emulsifiers, which has been used as a good alternative to Freund’s adjuvant for weak immunogens in animals [76]. The dose of CPS was determined according to the literature [34,46,55,77], as well as after dose-response experiments conducted with the purified *S. suis* type 2 CPS [54]. The placebo group received STIMUNE^®^ alone. On Days 7, 14, 21, 28, 35 and 42 post-immunization, blood samples were collected from the tail vein of each mouse for anti-CPS Ig titration.

In selected experiments aimed to evaluate the adjuvant effect of exogenous TLR agonists on the CPS-specific Ab response, mice were immunized s.c. with 2 µg of purified native or desialylated *S. suis* type 2 CPS in 100 µL of PBS on Day 0, followed by an administration of 80 µg of CpG 1826 ODNs (InvivoGen, San Diego, CA, USA) in 100 µL of PBS via the same route two days after. A group of mice similarly immunized with 2 µg of PS3 (ATCC 31-X), followed by 80 µg of CpG 1826 ODNs two days after, was included for comparative purpose. This immunization protocol was chosen based on the literature [34,46]. The control group received native or desialylated *S. suis* type 2 CPS or PS3 on Day 0 followed by PBS injection on Day 2. Two placebo groups receiving only PBS or CpG 1826 ODNs were also included. On Days 0 (before immunization), 7, 14 and 21 post-immunization, blood samples were collected from the tail vein of each mouse for anti-CPS Ig titration. On Day 5 [34,46], randomly selected mice of each group were euthanized, and spleens were removed to enumerate CPS-specific ASCs by enzyme-linked ImmunoSpot (ELISpot) (see below).

A last set of experiments aimed to evaluate the immunomodulatory effect of purified *S. suis* types 2, 14 and GBS types III and V CPSs on the development of the OVA-specific Ab response. On Day 0, mice were immunized i.p. with 10 µg of OVA (Sigma-Aldrich, Oakville, ON, Canada) in association with 2 µg of each individual native CPS in a final volume of 100 µL of PBS. A second dose with the same preparation was given on Day 21 post-primary immunization. Control group received OVA alone on Days 0 and 21. A placebo group receiving PBS alone was also included. On Days 7, 14, 21 and 35 post-immunization, blood samples were collected from the tail vein of each mouse for anti-OVA Ig titration.

The numbers of animals included in the experiments are detailed in the figure legends.

### 4.6. In Vitro B Cell Stimulation Assay

Untouched B cells were purified from the spleen of naive mice by negative selection using the B Cell Isolation Kit microbeads and magnetically activated cell sorting (MACS; Miltenyi Biotech, Auburn, CA, USA) according to the manufacturer’s instructions and resuspended at 10^6^ cells/mL in complete medium, consisting of RPMI 1640 supplemented with 10% heat-inactivated fetal bovine serum, 10 mM HEPES, 2 mM L-glutamine, 50 μM 2-mercaptoethanol, 100 U/mL penicillin-streptomycin and 20 µg/mL gentamycin (Gibco, Invitrogen, Burlington, ON, Canada). The enriched B cells had >95% purity as determined by fluorescence-activated cell sorter (FACS) analysis using anti-CD19 staining (data not shown).

To evaluate the immunomodulatory effect of purified *S. suis* types 2, 14 and GBS types III and V CPSs on the ability of BAFF or CpG 1826 ODNs to stimulate B cells, these cells were co-incubated with 20 µg/mL of each individual native or desialylated CPS and 1 µg/mL of BAFF along with 50 ng/mL of IL-4 (BioLegend, San Diego, CA, USA), as reported [64]. Similarly, in a second set of experiments, B cells were co-incubated with 1 µg/mL of CpG 1826 ODNs and 20 µg/mL of each individual native or desialylated CPS. After 7 days, supernatants were collected for quantification of the Ig secretion. In some experiments, B cells were pre-stimulated with CPS for 24 h prior to incubation with BAFF/IL-4 or CpG 1826 ODNs for 6 days. Cells stimulated with BAFF/IL-4 alone or CpG 1826 ODNs alone (without CPS) served as positive controls. In both set of experiments, the Ig secretion in the supernatants of B cells incubated with medium alone or each purified CPS alone was also measured as an indication of the basal Ig secretion by B cells in the absence of CpG 1826 ODNs or BAFF/IL-4 stimulation.

### 4.7. ELISA

#### 4.7.1. Samples from In Vivo Assays

For titration of protein-specific Abs in *S. suis*- or GBS-infected mice, Polysorb immunoplates (Canadawide Scientific, Toronto, ON, Canada) were coated with the respective non-encapsulated mutant strains as described previously [41]. Albeit a response to other cell wall components might be detected, proteins are the dominant antigens present at the surface of non-encapsulated mutants, and thus, the detected response in the ELISA is labelled as “anti-protein” throughout the study for simplicity. After washes in PBS containing 0.05% Tween 20 (PBS-T), mouse sera were serially diluted (two-fold) in PBS-T (starting with a dilution of 1/50) and incubated 1 h at room temperature (RT). After washes in PBS-T, plates were incubated with peroxidase-conjugated goat anti-mouse total Ig (IgG plus IgM), IgG (Jackson Immunoresearch, West Grove, PA, USA) or IgM (Southern Biotech, Birmingham, AL, USA) Abs for 1 h at RT. Plates were developed with 3,3′,5,5′-tetramethylbenzidine (TMB; Invitrogen) substrate, and the enzyme reaction was stopped by the addition of 0.5 M H_2_SO_4_. Absorbance was read at 450 nm with an ELISA plate reader. The reciprocal of the last serum dilution that resulted in an optical density (OD_450 nm_) equal or lower of 0.2 (cut-off) was considered the titer of that serum. When the OD_450 nm_ of the first dilution of a serum was lower than the cut-off, its titer was arbitrary fixed to 50. For titration of CPS- or OVA-specific Abs, a solution of 2 µg/mL of purified native *S. suis* type 2 or 14 or GBS type III or V CPS in carbonate buffer 0.1 M pH 9.6, or PS3 or OVA in PBS (100 µL/well) was added overnight on Polysorb immunoplates at 4 °C. Plates were then blocked 1 h at RT with 1% BSA in PBS before the addition of mouse serum dilutions. CPS- and OVA-specific Ab titers were determined as described above.

To control inter-plate variations, we added an internal reference positive control to each plate. For *S. suis* or GBS CPS- or protein-specific ELISA, this control was a pool of sera from mice hyperimmunized i.p. with 10^9^ CFU of heat-killed whole *S. suis* or GBS of the respective serotypes. For PS3-specific ELISA, this control was a pool of sera from mice immunized s.c. with 0.5 µg of PS3 followed by 80 µg of CpG 1826 ODNs two days after. For OVA-specific ELISA, this control was a pool of sera from mice co-immunized i.p. with 10 µg of OVA plus 20 µg of CpG 1826 ODNs. Reaction in TMB was stopped when an OD_450 nm_ of 1 was obtained for the positive control. Optimal dilutions of the coating antigen, the positive internal control sera and the peroxidase conjugated anti-mouse Abs were determined during preliminary standardizations.

#### 4.7.2. Samples from In Vitro Assays

The total IgM and IgG levels in B cell culture supernatants were measured with a Mouse IgM or a Mouse IgG ELISA Quantitation Set (Bethyl Laboratories, Montgomery, TX, USA), respectively, according to the manufacturer’s instructions. Two-fold dilutions of mouse reference IgM or IgG serum (Bethyl Laboratories) were used to generate standard curves. Sample dilutions giving OD readings in the linear portion of the appropriate standard curve were used to quantify the levels of Ig.

### 4.8. ELISpot

Spleens from mice immunized with native or desialylated *S. suis* type 2 or PS3 (with or without CpG 1826 ODNs) or from placebo groups (used as negative controls) were harvested 5 days post-immunization and pressed gently through a sterile fine wire mesh, as described above. After incubation with NH_4_Cl lysing buffer (eBioscience, San Diego, CA, USA) to remove red blood cells, total splenocytes were resuspended in complete medium. Assays were performed using 96-well MultiScreen high protein binding immunobilon-P membrane plates (Millipore, Billerica, MA, USA) coated with 2 µg/mL of purified native *S. suis* type 2 or PS3 in PBS, overnight at 4 °C. Plates were then washed in PBS and blocked for 2 h at 37 °C with complete medium. Splenocytes were serially diluted (two-fold) in complete medium (starting with a concentration of 5 × 10^6^ cells/mL) and 100 µL/well of each dilution was incubated on the plates for 24 h at 37 °C. Subsequently, plates were washed in PBS-T and incubated with peroxidase-conjugated goat anti-mouse total Ig (IgG plus IgM) Ab for 2 h at RT. Plates were developed with TMB substrate. Spots were counted using a CTL ImmunoSpot S4 ultraviolet Analyzer (Cellular Technology Limited, Cleveland, OH, USA). The background was subtracted, and data were expressed as the number of ASCs/10^6^ total splenocytes. Sample were considered positive if the number of ASCs was >10/10^6^ total splenocytes and 2 SD above the negative control. Optimal dilutions of the coating antigen and the peroxidase conjugate were determined during preliminary standardizations.

### 4.9. Statistical Analysis

Normality of data were evaluated using the Shapiro–Wilk test. Accordingly, the unpaired *t*-test (or the non-parametric Mann–Whitney rank sum test) was used in in vitro and in vivo studies. Analysis of variance (ANOVA) was used to analyze the significance of total anti-CPS Ab titers in Figure 1 and Figure 2, as well as the significance of Ab titers in Figures S2, S3, S6 and Figure A1. Data were analyzed with the Sigma Plot System (v11.0; Systat Software, Inc., San Jose, CA, USA). A *p* < 0.05 was considered as statistically significant.

## Supplementary Materials

The following are available online at www.mdpi.com/2076-0817/6/2/16/s1: Figure S1: Blood bacteremia of surviving mice after infection with *S. suis* serotype 2, *S. suis* serotype 14, GBS serotype III or GBS serotype V. Figure S2: Titration of CPS-specific antibodies in mice after immunization with purified native GBS serotype III. Figure S3: Titration of CPS-specific antibodies in mice after primary and secondary immunization with purified native or desialylated *S. suis* serotype 2, *S. suis* serotype 14, GBS serotype III or GBS serotype V CPS. Figure S4: Adjuvant effect of CpG ODNs on the CPS-specific antibody response in mice immunized with purified *S. pneumoniae* serotype 3 CPS (PS3). Figure S5: In vitro immunomodulatory effect of purified native or desialylated *S. suis* serotype 2, *S. suis* serotype 14, GBS serotype III or GBS serotype V CPS on the Ig secretion by naive B cells induced by CpG ODNs. Figure S6: Titration of CPS-specific antibodies in mice after immunization with purified native or desialylated *S. suis* serotype 2 CPS.

## Appendix A

In order to study the specific role of sialic acid linkage in the immunogenicity of GBS type III CPS, we substitute GBS type III α2,3-sialyltransferase *cpsK* gene (Accession Number AAD53072) by *S. suis* α2,6-sialyltransferase gene *cpsN* (Accession Number CAR45180), as recently described [78]. CPS expression in the α2,6 exchanged-GBS mutant was confirmed by a cell surface hydrophobicity test and transmission electron microscopy, as reported [10]. The CPS was purified as previously described [39] and subjected to rigorous physicochemical and quality control tests to ensure the identity and the purity of the CPS, as well as the presence of α2,6 sialic acid linkage instead of α2,3 linkage by nuclear magnetic resonance spectroscopy [78]. Purified CPS was used to immunize mice as described in the Materials and Methods (Section 4.5).
