# Supplementary material for: Evaluation of the Immunomodulatory Properties of Streptococcus suis and Group B Streptococcus Capsular Polysaccharides on the Humoral Response
Source: Pathogens. 2017 Apr 20;6(2):16. doi: 10.3390/pathogens6020016 (PMC5488650; doi:10.3390/pathogens6020016)
Supplement: Supplementary file 1 [file pathogens-06-00016-s001.pdf]

Supplementary Material

Evaluation of the immunomodulatory properties of *Streptococcus suis* and group B *Streptococcus* capsular polysaccharides on the humoral response

Cynthia Calzas [1], Morgan Taillardet [2], Insaf Salem Fourati [3], David Roy [1], Marcelo Gottschalk [1], Hugo Soudeyns [3], Thierry Defrance [2] and Mariela Segura [1,*]

[1] Swine and Poultry Infectious Diseases Research Center (CRIPA), Department of Pathology and Microbiology, Faculty of Veterinary Medicine, University of Montreal, 3200 Sicotte St., Saint-Hyacinthe, QC J2S 2M2, Canada;
[2] CIRI, INSERM, U1111, CNRS UMR5308, University of Lyon 1, 21 Avenue Tony Garnier, 69007 Lyon, France;
[3] Department of Microbiology, Infectiology and Immunology, Faculty of Medicine, University of Montreal, C.P. 6128, Succ. Centre-ville, Montreal, QC H3C 3J7, Canada;

* Correspondence: mariela.segura@umontreal.ca; Tel.: +1-450-773-8521 (ext. 0080)

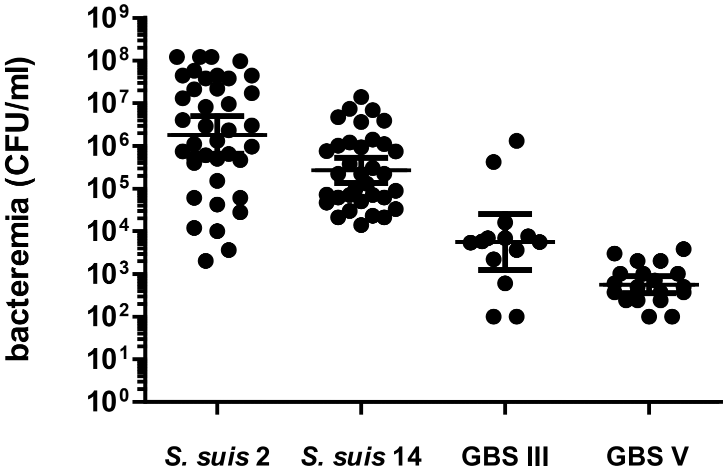

Figure S1. Blood bacteremia of surviving mice after infection with *S. suis* serotype 2, *S. suis* serotype 14, GBS serotype III, or GBS serotype V. Blood bacterial loads were determined 12 h post-infection of surviving mice after infection with 2 x 10^7 CFU of live *S. suis* serotype 2 strain P1/7 (60 mice were infected and 39 survived), 5 x 10^6 CFU of live *S. suis* serotype 14 strain DAN13730 (40 mice were infected and 34 survived), 2 x 10^6 CFU of live GBS serotype III strain COH-1 (40 mice were infected and 14 survived), or 10^4 CFU of live GBS serotype V strain CJB111 (40 mice were infected and 21 survived). Data from individual mice are presented, including the geometric mean with 95% confidence interval.

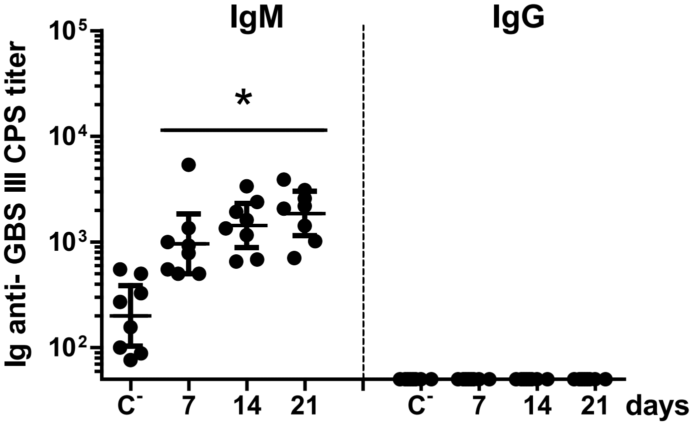

Figure S2. Titration of capsular polysaccharide (CPS)-specific antibodies in mice after immunization with purified native GBS serotype III CPS. Mice (n = 8) were immunized with 2 µg of purified native GBS serotype III CPS emulsified with STIMUNE®. IgM or IgG anti-native CPS titers were determined by ELISA on days 7, 14 and 21. "C⁻" represents a pool of control mice (n = 3) injected with STIMUNE® only, whose titers were evaluated on days 7, 14 and 21. Data from individual mice are presented, including the geometric mean with 95% confidence interval. *, statistically significant difference ($P < 0.05$) in comparison to the C⁻ group.

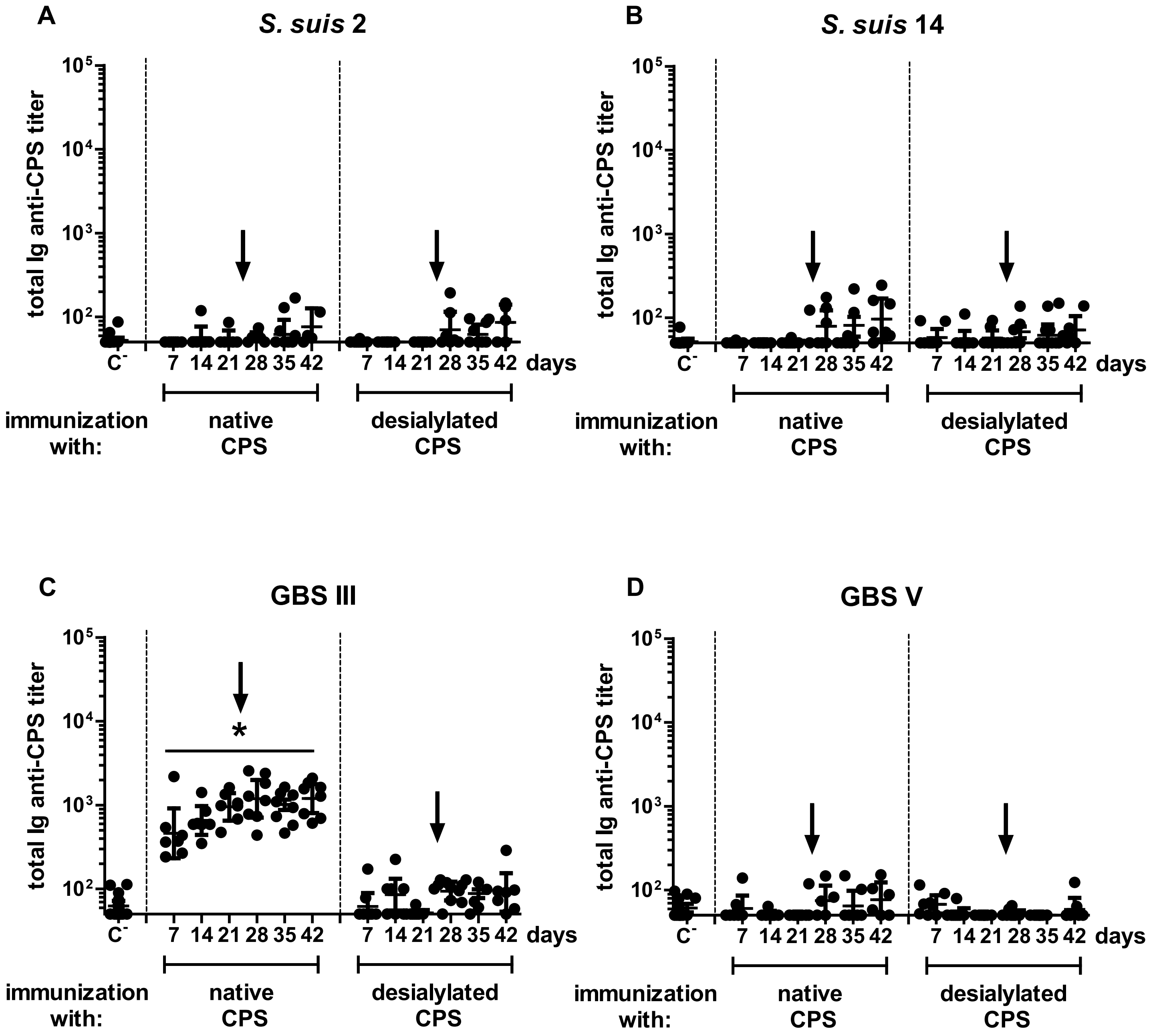

Figure S3. Titration of capsular polysaccharide (CPS)-specific antibodies in mice after primary and secondary immunization with purified native or desialylated *S. suis* serotype 2, *S. suis* serotype 14, GBS serotype III, or GBS serotype V CPS. Mice (n = 8) were immunized with 2 µg of purified native or desialylated *S. suis* serotype 2 (A), *S. suis* serotype 14 (B), GBS serotype III (C), or GBS serotype V (D) CPS emulsified with STIMUNE® on days 0 and 21. Total Ig (IgG plus IgM) anti-native CPS titers were determined by ELISA on days 7, 14, 21, 28, 35 and 42. "C^-" represents a pool of control mice (n = 3) injected with STIMUNE® only, whose titers were evaluated on days 7, 14, 21, 28, 35 and 42. Data from individual mice are presented, including the geometric mean with 95% confidence interval. An arrow indicates secondary immunization. *, statistically significant difference (P < 0.05) in comparison to the C^- group.

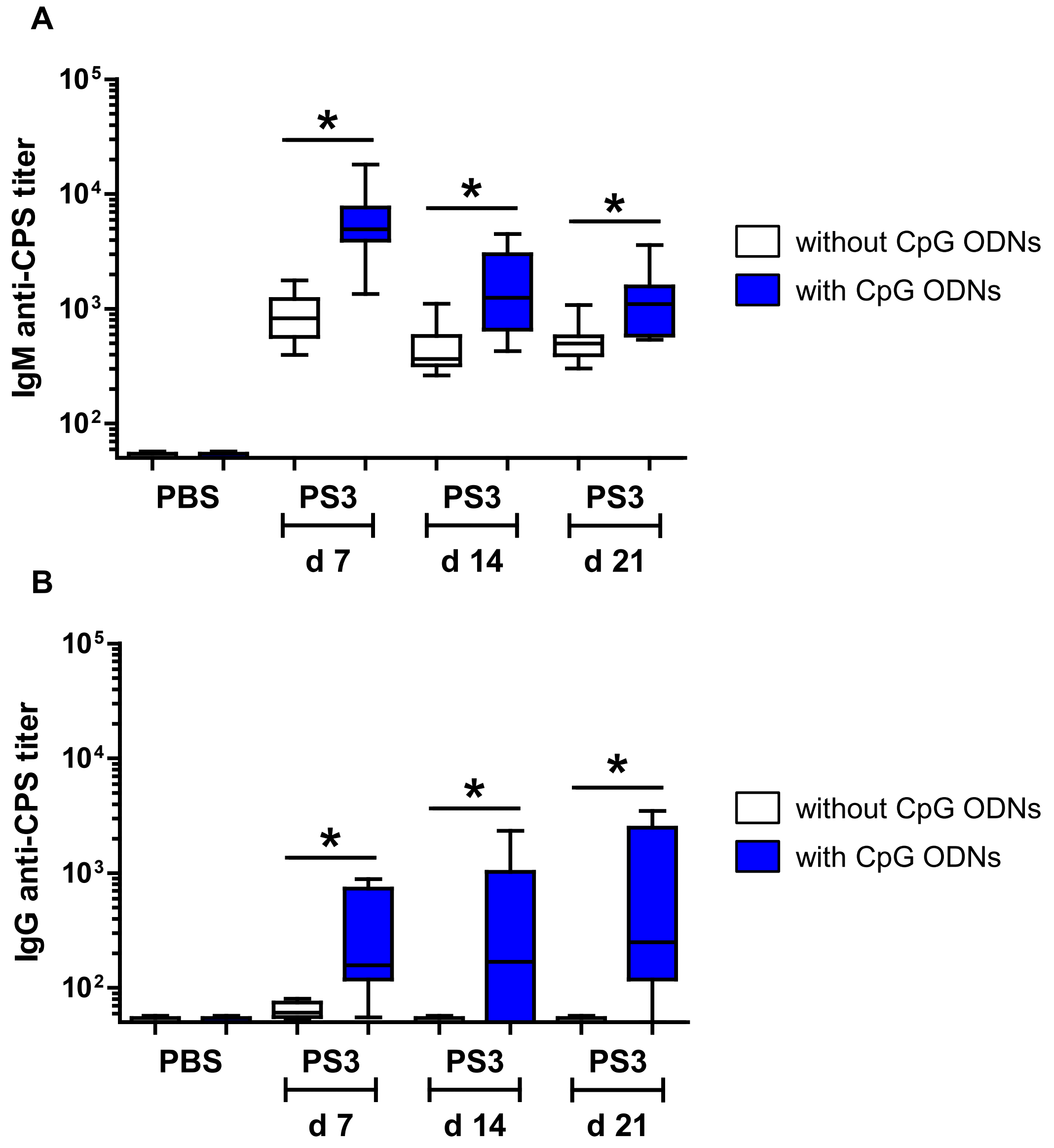

Figure S4. Adjuvant effect of CpG ODNs on the capsular polysaccharide (CPS)-specific antibody response in mice immunized with *S. pneumoniae* serotype 3 CPS (PS3). Mice (n = 8) were immunized with 2 µg of PS3 in PBS on day 0, and 80 µg of CpG ODNs two days after. Control group (n = 8) received PS3 on day 0, and PBS two days later. Placebo group (n = 3) received PBS or CpG ODNs only. IgM (A) and IgG (B) anti-PS3 titers were determined by ELISA on days 7, 14 and 21. Data are presented in a box-and-whisker diagram with ends of whiskers representing the minimum and the maximum value. *, $P < 0.05$ between “PS3” and “PS3 + CpG ODNs” groups.

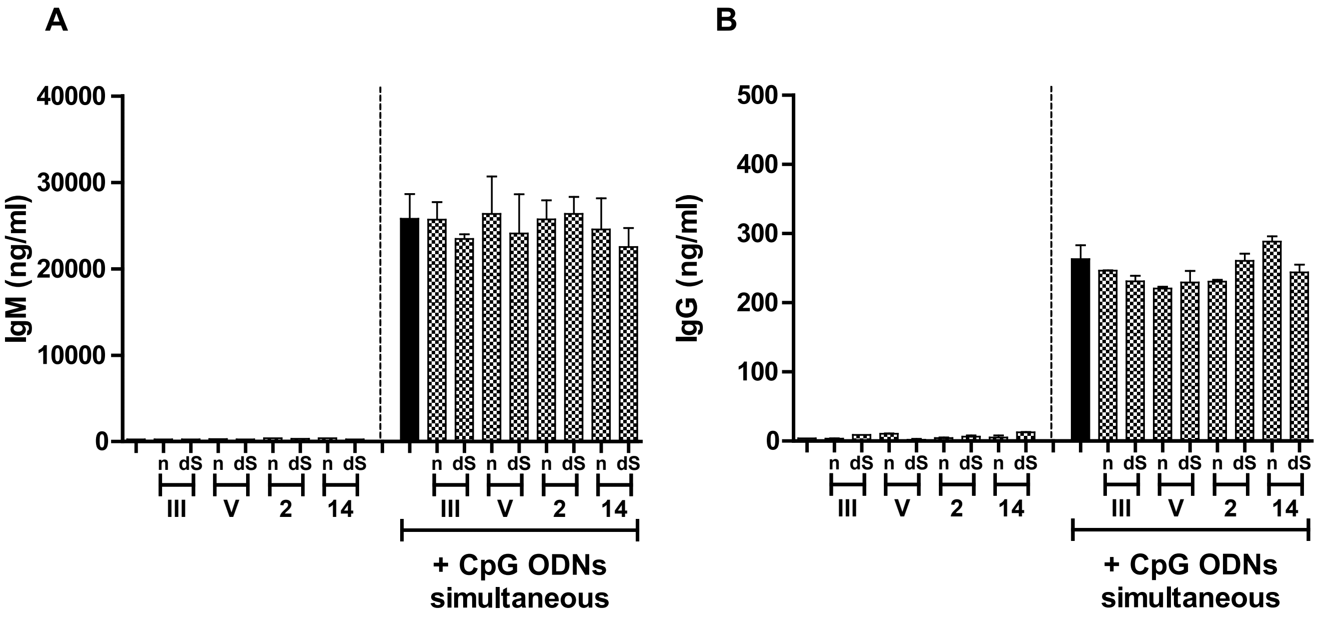

Figure S5. *In vitro* immunomodulatory effect of purified native or desialylated *S. suis* serotype 2, *S. suis* serotype 14, GBS serotype III, or GBS serotype V CPS on the Ig secretion by naïve B cells induced by CpG ODNs. Mouse splenic B cells (10^6 cells/ml) were incubated with purified native (n) or desialylated (dS) *S. suis* serotype 2, *S. suis* serotype 14, GBS serotype III, or GBS serotype V CPS (each at 20 µg/ml) simultaneously with CpG ODNs (1 µg/ml) (right panel). After 7 days of incubation, supernatants were collected and total IgM (A) and IgG (B) were quantified by ELISA. Cells stimulated with CpG ODNs alone (black bars) served as positive control. In left panel, cells stimulated with medium (white bars) or each purified CPS alone are represented as an indication of the basal Ig secretion level of cells in absence of stimulation by CpG ODNs. Data are expressed as arithmetic means with SEM of of three (right panel) or four (left panel) experiments.

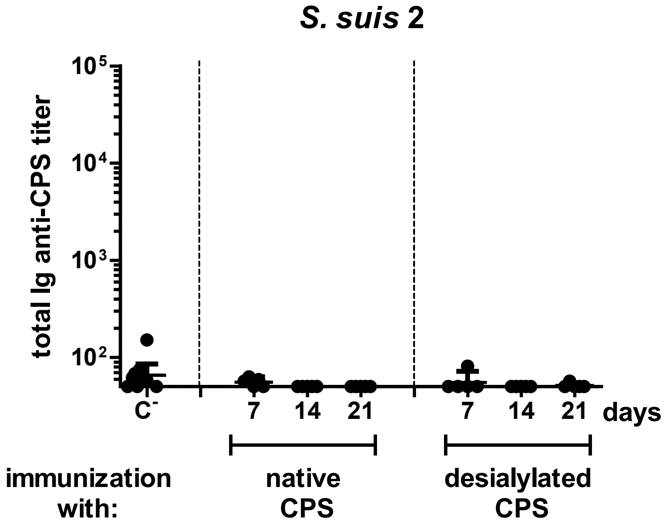

Figure S6. Titration of capsular polysaccharide (CPS)-specific antibodies in mice after immunization with purified native or desialylated *S. suis* serotype 2 CPS. Mice (*n* = 8) were immunized subcutaneously with 2 µg of purified native or desialylated *S. suis* serotype 2 CPS emulsified with the Sigma Adjuvant System® in a final volume of 200 µl (the adjuvant contains 0.5 mg monophosphoryl Lipid A - detoxified endotoxin- from *Salmonella minnesota* and 0.5 mg synthetic trehalose dicorynomycolate in 2% squalene oil-Tween® 80-water solution). Total Ig (IgG plus IgM) anti-native CPS titers were determined by ELISA on days 7, 14 and 21. "C-" represents a pool of control mice (*n* = 3) injected with the Sigma Adjuvant System ® only, whose titers were evaluated on days 7, 14 and 21. Data from individual mice are presented, including the geometric mean with 95% confidence interval.
